# Blanching influences the phenolics composition, antioxidant activity, and inhibitory effect of *Adansonia digitata* leaves extract on *α*‐amylase, *α*‐glucosidase, and aldose reductase

**DOI:** 10.1002/fsn3.386

**Published:** 2016-05-25

**Authors:** Emmanuel A. Irondi, Jacob K. Akintunde, Samson O. Agboola, Aline A. Boligon, Margareth L. Athayde

**Affiliations:** ^1^ Biochemistry UnitDepartment of Biosciences and BiotechnologyKwara State UniversityMalete, P.M.B. 1530IlorinNigeria; ^2^ Department of Veterinary Physiology, Biochemistry and PharmacologyUniversity of IbadanIbadanNigeria; ^3^ Phytochemical Research LaboratoryDepartment of Industrial PharmacyFederal University of Santa MariaBuilding 26, room 1115Santa MariaCEP 97105‐900Brazil

**Keywords:** *Adansonia digitata* leaves, blanching, carbohydrate‐hydrolyzing enzymes, diabetic complications, phenolics, type 2 diabetes

## Abstract

*Adansonia digitata* (*A*.* digitata*) leaves serve as food and has several medicinal uses in many parts of the world. This study evaluated the influence of blanching on the phenolics composition, antioxidant activity, and inhibitory effect of methanol extract of *A*.* digitata* leaves on the activities of some key enzymes (*α*‐amylase, *α*‐glucosidase, and aldose reductase) implicated in type 2 diabetes (T2D) in vitro. Reverse‐phase HPLC analysis revealed that the leaves had appreciable levels of flavonoids and phenolic acids, including catechin, epicatechin, rutin, quercitrin, quercetin, kaempferol, and luteolin (flavonoids); gallic, chlorogenic, caffeic, and ellagic acids (phenolic acids). Blanching caused significant (*P* < 0.05) decrease in the flavonoids and phenolic acids contents; DPPH* (2,2 diphenyl‐1‐picrylhydrazyl radical) and ABTS*^+^ [2,2‐azinobis (3‐ethyl‐benzothiazoline‐6‐sulfonic acid) radical cation] scavenging ability; reducing power; and Fe^2+^‐induced lipid peroxidation inhibitory capacity of the extract. Similarly, the inhibitory effect of the extract on the activities of *α*‐amylase, *α*‐glucosidase, and aldose reductase was significantly (*P* < 0.05) reduced due to blanching. Thus, *A*.* digitata* leaves extract could be effective for the management of T2D due to its flavonoids and phenolic acids content, antioxidant properties, and inhibitory potency on the activities of *α*‐amylase, *α*‐glucosidase, and aldose reductase. However, blanching militated against the levels of these functional attributes of the leaves and, therefore, may not be recommended for their optimal retention.

## Introduction

Type 2 diabetes (T2D) is a heterogeneous disorder characterized by hyperglycemia resulting from insulin resistance and the inability of pancreatic *β*‐cells to compensate for insulin resistance (DeFronzo 2004). The global prevalence of the disease is increasing alarmingly partly due to modern lifestyle and an increase in the consumption in high carbohydrate diets (Tappy and Le 2010), with its attendant postprandial hyperglycemia. Postprandial blood glucose level depends partly on the activities of carbohydrate‐hydrolyzing enzymes, mainly, *α*‐amylase and *α*‐glucosidase, that breakdown dietary starch and sugars into glucose (Sim et al. 2010); thereby making more glucose available for absorption. Uncontrolled diabetic hyperglycemia precipitates other factors that accelerate the progression of diabetic complications (Oishi et al. 2006), including the major microvascular complications – nephropathy, neuropathy, and retinopathy. Hence, the inhibition of carbohydrate‐hydrolyzing enzymes, with the resultant retardation of the digestion and absorption of dietary carbohydrate, is an important strategy for the prevention and management of T2D (Kwon et al. 2006; Oboh et al. 2010; Irondi et al. 2014). The inhibition of aldose reductase activity is also regarded as an important pharmacological strategy to prevent certain complications of diabetes (Saraswat et al. 2008).

Incidentally, acarbose and miglitol, the two leading oral hypoglycemic drugs that inhibit these enzymes have been reported to have some side effects such as diarrhea and other intestinal disturbances (Fujisawa et al. 2005), in addition to not being effective in maintaining euglycemia in diabetic patients. These limitations have necessitated more intensive research on plant products that could effectively reduce diabetic hyperglycemia and its associated complications possibly with less or no side effects (Ibrahim and Islam 2014). Interestingly, it has been suggested that about 90% of T2D cases could be possibly prevented by lifestyle modifications (Willett, 2002), including increased consumption polyphenolics‐rich plant foods.

*Adansonia digitata* L. (African baobab) is a deciduous tree indigenous to Africa where it is popularly referred to as the “tree of life” due to its ability to sustain life as a result of its water‐holding capacity, and its numerous ethnomedicinal and nutritional uses (Wickens and Lowe 2008). Various parts of the plant (including the leaves, pulp, fruits, seeds and bark) are sources of food, fiber and medicine for many people in Africa (Sibibe and Williams 2002; Chadare et al. 2009; De Caluwé et al. 2009). The various ethnomedicinal and food uses of *A*. *digitata* have attracted the interest of several pharmaceutical companies and researchers. Recently, the European Commission authorized the import of the fruit pulp as a novel food (Buchmann et al. 2010) and the Food and Drug Administration approved it as a food ingredient in the United States of America in 2009 (Addy 2009). In particular, the leaves of this plant which are eaten fresh or dried are the major source of food and folk medicine for many people in Africa (Ayele et al. 2013). For instance, the powdered leaves serve as a tonic and possess antihistamine and antitension properties. The leaves also possess antioxidant (Vertuani et al. 2002), anti‐inflammatory, and antiviral activities (Vimalanathan and Hudson 2009). They also have several other medicinal uses such as treatment of diseases of the urinary tract, guinea worm, internal pains, dysentery, and ophthalmia (Sibibe and Williams 2002). Methanol and aqueous extracts of the leaves of the plant have been reported to contain glycosides, phytosterols, saponins, protein and amino acid, phenolic compounds and tannins, gums, mucilage, and flavanoids (Shri et al. 2004).

Blanching, as a vegetable processing method, involves the heating of the vegetables to a temperature sufficiently high to destroy the enzymes present in the tissue. It is a popular method of processing vegetables, including *A*.* digitata* leaves, that is intended to terminate the action of enzyme, shorten the drying and dehydration time, and set the color of the vegetables (Wen et al. 2010). However, how it impacts the phenolics composition, antioxidant activity, and inhibitory effect of *A*.* digitata* leaves extract on some enzymes associated with T2D has not been reported. This study was therefore designed to evaluate the influence of blanching on the phenolics composition, antioxidant activity, and inhibitory effect of *A*.* digitata* leaves extract on *α*‐amylase, *α*‐glucosidase, and aldose reductase in vitro.

## Materials and Methods

### Collection and preparation of samples

About 200 g of fresh leaves sample was randomly collected from *A*.* digitata* tree in a local farm settlement in Akingbile area of Moniya, Ibadan, Nigeria (Figure S1). The sample was authenticated at the Department of Botany, University of Ibadan, Nigeria. After sorting, the sample was divided into two portions; a portion was blanched, whereas the second portion was not. Blanching was carried out by immersing 50 g of whole Baobab leaves in 5 L of hot water (80°C) in a thermostatically regulated water bath (±0.5°C) for 10 min, using a wire mesh basket. After blanching, the basket was taken out and the water retained in the leaves was drained off using a plastic kitchen sieve.

Then the leaves were allowed to cool and dry under air‐conditioner for 7 days. The raw leaves were also dried under the same condition. After drying, the samples were milled into fine particle size (0.5 mm), packed in air‐tight plastic vials, and stored at −4°C until analysis.

### Preparation of extract

Two gram of the sample was extracted by steeping in 100 mL of methanol overnight, after which it was filtered through Whatman No. 1 filter paper. The filtrate, subsequently referred to as extract, was stored at −4°C for further analysis.

### Handling of experimental animal

Adult male Wister strain albino rats weighing 200–250 g were procured from the experimental animal breeding unit of Department of Veterinary Medicine, University of Ibadan, Nigeria. The rats were handled in accordance with our institution guide for the care and use of laboratory animals. The rats were kept in a cage to acclimatize for 7 days, during which they were maintained on a 12 h light/12 h dark cycle at 25°C, with free access to food and water.

### Quantification of phenolic compounds by HPLC‐DAD

Sample extracts at a concentration of 15 mg/mL were injected by means of Auto‐sampler (Shimadzu, model SIL‐20A, Kyoto, Japan). Separations were carried out using Phenomenex C_18_ column (4.6 mm x 250 mm × 5 *μ*m particle size). The mobile phase comprised solvent A [water: formic acid (98:2, v/v)] and solvent B (acetonitrile), at a flow rate of 0.6 mL/min and injection volume of 40 *μ*L. Gradient program was started with 95% of A and 5% of B until 2 min and changed to obtain 25%, 40%, 50%, 70%, and 80% B at 10, 20, 30, 50, and 70 min, respectively, following the method described by Boligon et al. (2015), with slight modifications. The sample and mobile phase were filtered through 0.45 *μ*m membrane filter (Millipore, USA) and then degassed by ultrasonic bath prior to use. Stock solutions of standards references were prepared in the HPLC mobile phase at a concentration range 0.025–0.300 mg/mL. Quantifications were carried out by integration of the peaks using the external standard method, at 254 nm for gallic acid and ellagic acid; 280 nm for catechin and epicatechin; 325 nm for caffeic acid and chlorogenic acid; and 366 for quercetin, quercitrin, kaempferol, luteolin, and rutin. The chromatography peaks were confirmed by comparing its retention time with those of reference standards and by DAD spectra (200–600 nm). Calibration curve for gallic acid: *Y* = 13581*x* + 1195.7 (*r* = 0.9997); catechin: *Y* = 12609x + 1187.4 (*r* = 0.9996); epicatechin: *Y* = 12719*x* + 1356.9 (*r* = 0.9995); caffeic acid: *Y* = 12750x + 1352.9 (*r* = 0.9999); chlorogenic acid: *Y* = 11825*x* + 1383.6 (*r* = 0.9998); ellagic acid: *Y* = 13192*x* + 1176.5 (*r* = 0.9997); quercitrin: *Y* = 12641*x* + 1295.7 (*r* = 0.9999); kaempferol: *Y* = 11846x + 1283.9 (*r* = 0.9994); rutin: *Y* = 11792*x* + 1305.8 (*r* = 0.9995); luteolin: *Y* = 13894*x* + 1267.1 (*r* = 0.9998); and quercetin: *Y* = 12618*x* + 1196.3 (*r* = 0.9996). All chromatography operations were carried out at ambient temperature and in triplicate.

### LOD and LOQ of phenolic compounds

Limit of detection (LOD) and limit of quantification (LOQ) were calculated based on the standard deviation of the responses and the slope using three independent analytical curves, as defined by Khaliq et al. (2015). LOD and LOQ were calculated as 3.3 and 10 *σ*/S, respectively, where *σ* is the standard deviation of the response and S is the slope of the calibration curve.

### Determination of ABTS*^+^ scavenging ability

The ABTS*^+^[2,2‐azinobis (3‐ethyl‐benzothiazoline‐6‐sulfonic acid) radical cation] scavenging ability of the extracts was determined according to the method described by Sellappan and Akoh (2002). The ABTS*^+^ was generated by incubating equal volume of 7 mmol/L ABTS*^+^ aqueous solution with potassium persulfate (2.45 mmol/L) in the dark for 16 h at room temperature and adjusting the absorbance at 734 nm to 0.7 ± 0.02 with 95% ethanol. Then 0.2 mL appropriate dilution of the extract was added to 2.0 mL ABTS*^+^ solution and the absorbance were measured at 734 nm after 15 min. The ABTS*^+^ scavenging ability of the extracts was subsequently calculated and expressed in trolox equivalent (TE).

### Determination of reducing power

The reducing power of the extracts was determined by assessing their ability to reduce FeCl_3_ solution as described by Oyaizu (1986). An aliquot of 2.5 mL extract was mixed with 2.5 mL of 200 mmol/L sodium phosphate buffer (pH 6.6) and 2.5 mL of 1% potassium ferricyanide. The reaction mixture was incubated at 50°C for 20 min and then 2.5 mL of 10% trichloroacetic acid was added. This mixture was centrifuged at 650*g* for 10 min. Then 5 mL supernatant was mixed with an equal volume of water and 1 mL of 0.1% ferric chloride. The absorbance was measured at 700 nm. The reducing power of the extracts was subsequently calculated and expressed in gallic acid equivalent (GAE).

### Determination of DPPH free radical scavenging ability

The DPPH* scavenging ability of the extracts was determined as described by Cervato et al. (2000). Briefly, different concentrations (0.10, 0.20, 0.30, and 0.40 mg/mL) of the extracts amounting to 1 mL were mixed with 3 mL of 60 *μ*mol/L DPPH*; the mixture was left in the dark for 30 min before the absorbance was taken at 517 nm. A reference test (DPPH* solution without the extract) was included in the assay. The percentage DPPH* scavenging ability of the extract was calculated using the formula: % scavenging ability = [(A517_reference_ − A517_sample_)/A517_reference_] × 100; where A517_reference_ is the absorbance of the reference test; and A517_sample_ is the absorbance of the test containing the extract.

### Inhibition of lipid peroxidation assay

The ability of the extracts to inhibit Fe^2+^‐induced lipid peroxidation in rat pancreas homogenate was assayed according to the modified method of Ohkawa et al. (1979). The rats were mildly anesthetized in ether, and their pancreas was rapidly excised and placed in ice. Then 10% (w/v) homogenate was prepared by homogenizing the pancreas in cold saline, and centrifuging for 10 min at 1400*g* to yield a low‐speed supernatant (LSS) that was used for the assay. To a reaction mixture containing 100 *μ*L of the LSS, 30 *μ*L of 0.1 mol/L Tris‐HCl buffer (pH 7.4) and different concentrations (0.10, 0.20, 0.30, and 0.40 mg/mL) of leaves extract, and 30 *μ*L of freshly prepared 25 *μ*mol/L ferric sulfate solution were added to initiate lipid peroxidation. The volume was made up to 300 *μ*L with deionized water before incubation at 37°C for 1 h. The color reaction was initiated by adding 300 *μ*L of 81 g/L sodium duodecyl sulfate to the reaction mixture containing the LSS, followed by the addition of 600 *μ*L of acetic acid/HCl (pH 3.4) and 600 *μ*L of 0.8% (v/v) TBA (Thiobarbituric acid). This mixture was incubated at 100°C for 1 h. The absorbance of thiobarbituric acid reactive species (TBARS) produced was measured at 532 nm in a UV‐visible spectrophotometer. A reference test not containing the plant extract was carried out so as to obtain the absorbance of the maximum TBARS formed. The percentage inhibition of Fe^2+^‐induced lipid peroxidation by the extract was calculated using the formula: % Inhibition = [(A532_reference_ − A532_sample_)/A532_reference_] × 100; where A532_reference_ is the absorbance of the reference test; and A532_sample_ is the absorbance of the test containing the extract.

### Alpha‐amylase inhibition assay

Alpha‐amylase inhibition assay was carried out following the procedure described by Kwon et al. (2008). Appropriate dilutions (0–200 *μ*L) of the extracts amounting to 500 *μ*L, and 500 *μ*L of 0.02 mol/L sodium phosphate buffer (pH 6.9 with 0.006 mol/L sodium chloride) containing 0.5 mg/mL *α*‐amylase solution (porcine pancreas *α*‐amylase; Sigma, Cat. No. A3176, Chemical Co., St. Louis, MO, USA), were incubated at 37°C for 10 min. After preincubation, 500 *μ*L of 1% starch solution in 0.02 mol/L sodium phosphate buffer was added. The reaction mixture was then incubated at 37°C for 15 min, and the reaction was terminated with 1.0 mL of DNSA color reagent (1% 3, 5‐dinitrosalicylic acid and 12% sodium potassium tartrate in 0.4 mol/L NaOH). The reaction mixture was then incubated in a boiling water bath for 5 min, cooled to room temperature, and diluted with 10 mL distilled water. A reference test (without the plant extract) and a negative control test were carried out. The absorbance was measured at 540 nm. Percentage *α*‐amylase inhibition was calculated using the formula: % Inhibition = [(A540_reference_ − A540_sample_)/A540_reference_] × 100; where A540_reference_ is the absorbance of the reference test; and A540_sample_ is the absorbance of the test containing the extract.

### Αlpha‐glucosidase inhibition assay

The inhibitory effect of extracts against *α*‐glucosidase activity was determined according to the method described by Kim et al. (2005), using *α*‐glucosidase from *B. stearothermophillus* (Sigma, Cat. No. G 3651). Briefly, *α*‐glucosidase (5 units) was preincubated with different dilutions (0–200 *μ*L) of the extracts for 15 min. Next, 3 mmol/L para‐nitrophenylglucopyranoside (PNPG) dissolved in 20 mmol/L phosphate buffer (pH 6.9) was added to start the reaction. The reaction mixture was further incubated at 37°C for 20 min, after which 2 mL of 0.1 mol/L Na_2_CO_3_ was added to stop the reaction. The absorbance of the yellow colored p‐nitrophenol released from PNPG was measured at 400 nm. A reference test (without the plant extract) and a negative control test were carried out, and the percentage of *α*‐glucosidase inhibition was calculated as follows: % Inhibition = [(A400_reference_ − A400_sample_)/A400_reference_] × 100; where A400_reference_ is the absorbance of the reference test; and A400_sample_ is the absorbance of the test containing the extract.

### Aldose reductase inhibition assay

Partially purified rat lens aldose reductase was prepared following a procedure adapted from Hayman and Kinoshita (1965). Briefly, lenses were quickly removed from rats following anesthesia with ether, and homogenized in a glass homogenizer with a Teflon pestle in five volume of ice‐cold distilled water. The homogenate was centrifuged at 10,000*g* at 0–4°C for 20 min. The supernatant was precipitated with saturated ammonium sulfate at 40%, 50%, and then at 75% salt saturation. The supernatant was retained after the first two precipitations. The pellet from the last step, possessing aldose reductase activity, was dispersed in 75% ammonium sulfate and subsequently used for the inhibition assay.

Inhibition of AR activity was assayed by estimating the consumption of NADPH at 340 nm as described by Da Settimo et al. (2005). The reaction mixture contained different dilutions (0–200 *μ*L) of the extracts, 4.67 mmol/L D,L‐glyceraldehyde as substrate, 0.11 mmol/L NADPH, 0.067 mol/L phosphate buffer (pH 6.2), and 0.05 mL of the enzyme preparation (rat lens homogenate) in a total volume of 1.5 mL. The enzyme reaction was initiated by addition of D,L‐glyceraldehyde and was monitored for 4 min after an initial period of 1 min at 30°C. A reference test (without the plant extract) and a negative control test were carried out. A decrease in absorbance of sample test relative to the reference test at 340 nm is a function amount of NADPH consumed. Hence, % Inhibition = [(A340_reference_ − A340_sample_)/A340_reference_] × 100; where A340_reference_ is the absorbance of the reference test; and A340_sample_ is the absorbance of the test containing the extract.

### Statistical analysis

Results of triplicate experiments were expressed as mean ± standard deviation (SD). *T*‐test for independent samples was carried out on the result data at 95% confidence level using SPSS statistical software package, version 17, (SPSS Inc., Chicago). Half‐maximal inhibitory concentration (IC_50_) and half‐maximal scavenging concentration (SC_50_) were calculated from the % inhibition versus extract concentration nonlinear regression curve of each extract.

## Results

### Phenolics composition

**Figure 1:**
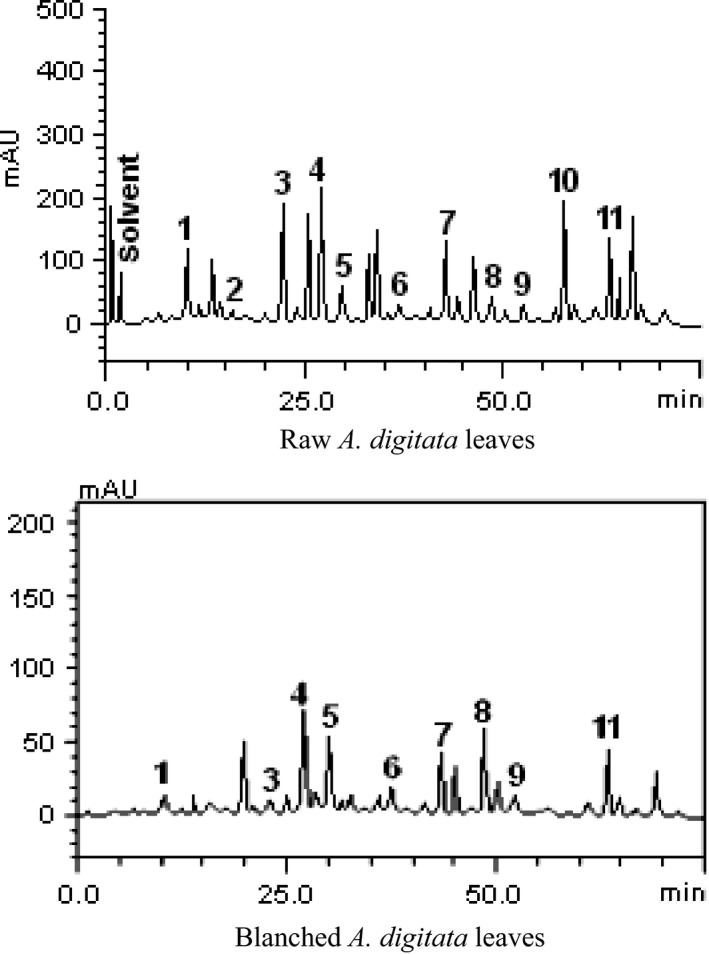
Representative high‐performance liquid chromatography profile of the extracts. UV detection was at 325 nm. Gallic acid (peak 1), catechin (peak 2), chlorogenic acid (peak 3), caffeic acid (peak 4), ellagic acid (peak 5), epicatechin (peak 6), rutin (peak 7), quercitrin (peak 8), quercetin (peak 9), kaempferol (peak 10), and luteolin (peak 11). Chromatographic conditions are described in the Materials and Methods section.

The HPLC fingerprinting of the raw *A*.* digitata* leaves extract (Fig. 1) revealed the presence of gallic acid (Retention time, R_*t*_ = 10.19 min; peak 1), catechin (R_*t*_ = 16.07 min; peak 2), chlorogenic acid (R_*t*_ = 22.35 min; peak 3), caffeic acid (R_*t*_ = 26.97 min; peak 4), ellagic acid (R_*t*_ = 29.83 min; peak 5), epicatechin (R_*t*_ = 37.41 min; peak 6), rutin (R_*t*_ = 43.86 min; peak 7), quercitrin (R_*t*_ = 48.13 min; peak 8), quercetin (R_*t*_ = 52.01 min; peak 9), kaempferol (R_*t*_ = 57.69 min; peak 10), and luteolin (R_*t*_ = 64.15 min; peak 11). In contrast, the HPLC fingerprinting of the blanched leaves showed the presence of all the phenolic compounds found in the raw leaves except catechin and kaempferol that were not detected (Fig. 1).

Table 1 shows the phenolics composition of the raw and blanched leaves. Generally, the raw leaves had appreciable levels of the phenolics. The levels of the flavonoids were in the order of kaempferol > luteolin > quercetrin > quercetin > rutin > epicatechin > catechin; whereas the levels of the phenolic acids were in the order of chlorogenic acid > caffeic acid > gallic acid > ellagic acid. Blanching led to significant (*P* < 0.05) losses in the levels of both the flavonoids (except quercetin) and phenolic acids. Among the flavonoids, blanching resulted in 100% loss of catechin and kaempferol, as they were not detected in the blanched leaves. Interestingly, blanching led to 45.4% increase in the level of quercetin. Similarly, there were losses in the levels of the phenolic acids due to blanching; with chlorogenic acid (91.88% loss) and gallic acid (86.51% loss) being the most affected.

**Table 1:** Phenolics composition of raw and blanched *A. digitata* leaves

Compounds (mg/g)	Raw	Blanched	LOD *μ*g/mL	LOQ *μ*g/mL	% Change
Gallic acid	4.15 ± 0.01^a^	0.56 ± 0.02^b^	0.024	0.079	−86.51
Catechin	0.75 ± 0.01	ND	0.013	0.042	−100.00
Chlorogenic acid	6.28 ± 0.03^a^	0.51 ± 0.03^b^	0.008	0.026	−91.88
Caffeic acid	6.31 ± 0.02^a^	2.79 ± 0.01^b^	0.019	0.061	−55.78
Ellagic acid	2.43 ± 0.01^a^	2.34 ± 0.01^a^	0.026	0.085	−3.70
Epicatechin	0.79 ± 0.04^a^	0.55 ± 0.04^b^	0.011	0.034	−30.38
Quercitrin	4.18 ± 0.01^a^	2.13 ± 0.01^b^	0.020	0.066	−49.04
Quercetin	1.63 ± 0.01^b^	2.37 ± 0.02^b^	0.027	0.089	45.40
Rutin	0.81 ± 0.03^a^	0.53 ± 0.01^b^	0.014	0.047	−34.57
Kaempferol	6.29 ± 0.02	ND	0.008	0.026	−100.00
Luteolin	4.54 ± 0.01^a^	2.30 ± 0.02^b^	0.017	0.056	−49.34

Results are expressed as mean ± standard deviations (SD) of three determinations. Values followed by different superscript letters in each row differ significantly at *P* < 0.05.

LOD, Limit of detection; LOQ, Limit of quantification.

The antioxidant activity of the raw and blanched leaves was tested using four different assays, namely, ABTS*^+^ and DPPH* scavenging assays, reducing power, and lipid peroxidation inhibition assays. The results, presented in Table 2, revealed that both the raw and blanched leaves extracts were able to scavenge ABTS*^+^ and DPPH*. The ABTS*^+^ scavenging ability of the raw leaves extract (1.87 ± 0.09 mmol/L TE/g) was significantly (*P* < 0.05) higher than that of the blanched leaves extract (1.43 ± 0.06 mmol/L TE/g). The DPPH* SC_50_ of the raw leaves extract (0.23 ± 0.01 mg/mL) was significantly (*P* < 0.05) lower than that of the blanched leaves extract (0.30 ± 0.02 mg/mL), indicating that the raw leaves extract had stronger DPPH* scavenging ability than the blanched leaves extract. Similarly, the raw leaves extract had significantly (*P* < 0.05) higher reducing power than the blanched leaves extract. Extracts of both the raw and blanched leaves inhibited Fe^2+^‐induced lipid peroxidation in rat pancreas homogenate; however, the raw leaves (IC_50_: 0.18 ± 0.01 mg/mL) had more inhibitory potency than the blanched leaves (IC_50_: 0.27 ± 0.01 mg/mL).

**Table 2:** ABTS*^+^ scavenging ability, reducing power, DPPH SC_50_, and Lipid peroxidation IC_50_ of raw and blanched *A. digitata* leaves extract

Treatment	ABTS*^+^ scavenging ability (mM TE/g)	Reducing power (mg GAE/g)	DPPH SC_50_ (mg/mL)	Lipid peroxidation IC_50_ (mg/mL)
Raw	1.87 ± 0.09^a^	20.02 ± 1.83^a^	0.23 ± 0.01^b^	0.18 ± 0.01^b^
Blanched	1.43 ± 0.06^b^	16.80 ± 1.02^b^	0.30 ± 0.02^a^	0.27 ± 0.01^a^

Results are expressed as mean ± standard deviations (SD) of replicate determinations. Values followed by different superscript letters in each column differ significantly at *P* < 0.05.

ABTS*^+^, [2,2‐azinobis (3‐ethyl‐benzothiazoline‐6‐sulfonic acid) radical cation]; GAE, gallic acid equivalent.

The half‐maximal inhibitory concentrations (IC_50_) of the raw and blanched leaves extracts on *α*‐amylase, *α*‐glucosidase, and aldose reductase activities are presented in Table 3. The raw and blanched leaves were both able to inhibit the activities of these three carbohydrate‐metabolizing enzymes that are implicated in T2D. However, the raw leaves extract was more potent in inhibiting the activities of these enzymes, as indicated by its lower IC_50_ values in comparison with those of the blanched leaves extract. Aldose reductase was the most inhibited, followed by *α*‐glucosidase and *α*‐amylase.

**Table 3:** IC_50_ of raw and blanched *A. digitata* leaves extracts on *α*‐amylase, *α*‐glucosidase, and aldose reductase activities

Treatment	*α*‐amylase IC_50_ (mg/mL)	*α*‐glucosidase IC_50_ (mg/mL)	Aldose reductase IC_50_ (mg/mL)
Raw	1.18 ± 0.04^b^	0.55 ± 0.01^b^	0.43 ± 0.01^b^
Blanched	1.79 ± 0.12^a^	0.92 ± 0.01^a^	0.87 ± 0.02^a^

Results are expressed as mean ± standard deviations (SD) of replicate determinations. Values followed by different superscript letters in each column differ significantly at *P* < 0.05.

## Discussion

Although blanching is intended to terminate the action of enzyme, shorten the drying and dehydration time, and set the color of vegetables (Wen et al. 2010), studies have shown that it influences the functional attributes of several plant products including the polyphenol content of *Coriandrum sativum* leaves and fruits (Kaiser et al. 2013); the total phenolics content and antioxidant activity of turnip greens (Martínez et al. 2013); the color, texture, polyphenols, and antioxidant capacity of Irish York cabbage (Jaiswal et al. 2012); and the inhibitory ability of A*maranthus cruentus* extract on *α*‐amylase and *α*‐glucosidase activities (Oboh et al. 2013). Hence, the influence of blanching on the phenolics composition, antioxidant activity, and inhibitory effects of *A*.* digitata* leaves extract on the activities of *α*‐amylase, *α*‐glucosidase, and aldose reductase was evaluated in this study.

The phenolic compounds quantified in the leaves fall into two important classes of phenolics, namely, flavonoids and phenolic acids. The flavonoids include catechin, epicatechin, quercetrin, quercetin, rutin, kaempferol, and leuteolin; whereas the phenolic acids include gallic, chlorogenic, caffeic, and ellagic acids. Flavonoids are prominent as both electrons donors and terminators of chain reactions due to their hydroxyl groups, particularly at the 3′OH and 4′OH of their three‐carbon chain (Kim et al. 2010; Cho et al. 2013). In contrast, phenolic acids contain a phenolic ring and an organic carboxylic acid function (Goufo et al. 2014), and the phenolic ring are capable of stabilizing and delocalizing unpaired electrons (Goufo and Trindade 2014). These distinguishing structural features confer antioxidant property on flavonoids and phenolic acids. Flavonoids and phenolic acids are also of pharmacological importance for the management and/or prevention of T2D (Ghasemzadeh and Ghasemzadeh 2011). The raw leaves proved to be a rich source of these two classes of phenolics. Blanching resulted in significant (*P* < 0.05) losses in both the flavonoids (except quercetin) and phenolic acids content of the leaves; with ellagic acid (3.70% loss) as the least affected, and catechin and kaempferol (100% loss) as the most affected. Loss in the phenolics content of vegetables due to blanching has been reported by some previous studies (Sikora et al. 2008; Korus and Lisiewska 2011; Jaiswal et al. 2012), and this could be attributed to oxidation of the phenolics or their leaching into water during blanching (Roy et al. 2009). Phenolic compounds are known to occur in soluble forms and in combination with cell wall components in plants (Francisco et al. 2010). Thus, the high temperature of the blanching may have led to the disruption of the cell walls and the breakdown of the phenolics, thereby leading to their leaching into the blanching water. The differential losses in the levels of the individual flavonoids and phenolic acids after blanching may reflect variations in their individual solubility and thermo‐stability in hot water. Francisco et al. (2010) have earlier demonstrated that the same method of cooking affects different types of flavonoids even within the same class differently. Thus, the absolute loss (100%) in catechin and kaempferol may suggest that these two flavonoids are highly water soluble and heat labile. Against the losses recorded for all other phenolics, the level of quercetin increased by 45.4% after blanching. This exclusive increase may be attributed to enhanced extractability (Wen et al. 2010), following the disruption of the cell wall of the leaves, as well as the hydrolysis of quercitrin.

The extracts scavenged DPPH* and ABTS*^+^, and reduced Fe^3+^ to Fe^2+^ (Table 2), and these represent their antioxidant activity. This finding corroborates previous reports that *A. digitata* leaves extract posses antioxidant activity (Vertuani et al. 2002; Ayele et al. 2013). Blanching attenuated the antioxidant activity of the leaves; this is also in agreement with several earlier studies that demonstrated that blanching reduces the antioxidant activity of vegetables (Korus and Lisiewska 2011; Jaiswal et al. 2012). This negative effect has been attributed to the loss in the phenolics and other water soluble antioxidants due to leaching and thermal degradation during blanching. However, Roy et al. (2009) noted that the influence of thermal processing on polyphenolics level and antioxidative activity is dependent on the type of product. In addition, Sikora et al. (2008) noted that the extent of polyphenolics degradation is very much dependent on processing time and the size of vegetables. Hence, Wen et al. (2010) reported that after blanching, the total phenolic content and antioxidant activity of five commonly consumed vegetables in Malaysia either decreased or increased depending on the type of vegetables.

Free radicals and reactive oxygen species (ROS) are notorious for the oxidative damage they cause to intra‐ and extracellular macromolecules, including damaging of the DNA, protein, lipids, and carbohydrates (Takemoto et al. 2009). Oxidative stress is induced when the free radicals and ROS (oxidants) burden of the cell overwhelms its antioxidant defense system, and it has been implicated in the pathogenesis and complications of diabetes (Giacco and Brownlee 2010). Lipid peroxidation, which is the oxidative damage of lipids (particularly, the polyunsaturated fatty acids that are very vulnerable to oxidative attack) by ROS and transition metal ions, also plays a major role in cell injury (Schafer et al. 2000), as it yields diverse cytotoxic products, mostly aldehydes such as malondialdehyde (MDA) (Shalaby and Shanab 2013). The toxic effects of these products are due to their ability to disrupt the function of membrane; inactivate membrane‐bound receptors and enzymes, and compromise membrane permeability (Rahman 2003). As a chain reaction, lipid peroxidation in turn propagates the production of more free radicals that can induce oxidative damage to the body cells, including the *β*‐cells of the pancreas (Shah and Fonseca 2011). This explains why tissue damages mediated by lipid peroxides have been observed in diabetes (Feillet‐Coudray et al. 1999). However, these oxidative damages could be mitigated by a robust cellular antioxidant defense system. This therefore means that increasing the intake of *A*.* digitata* leaves rich in phenolics could prevent the onset of oxidative stress, and ameliorate its sequelae. Furthermore, as the leaves extract possesses reducing power, as seen in its reduction of Fe^3+^ to Fe^2+^, it could reduce the oxidized intermediates of lipid peroxidation by acting as electron donor, thereby functioning as primary and secondary antioxidants (Chanda and Dave 2009). However, as in the radical scavenging results, blanching reduced the ability of the extract to inhibit Fe^2+^‐induced lipid peroxidation in rat's pancreas homogenate.

Plant foods rich in phenolics are important for the management and treatment of T2D. Studies have consistently shown that the inhibition of carbohydrate‐hydrolyzing enzymes, including *α*‐amylase, *α*‐glucosidase, and aldose reductase, is one of the possible mechanisms through which plant‐derived phenolics exhibit their antidiabetic activity (Adefegha and Oboh 2012; Irondi et al. 2014). Indeed, the hydrolytic activity of amylase and glucosidase on carbohydrates contributes to postprandial blood glucose level (Sim et al. 2010). Hence, by inhibiting *α*‐amylase and *α*‐glucosidase, with the concomitant decrease in the rate of glucose absorption, the phenolics in *A. digitata* leaves extract can reduce the rise in postprandial blood glucose. Interestingly, the inhibition of *α*‐amylases and *α*‐glucosidases activities has been reported as one of the most effective approaches to control hyperglycemia in T2D (Kim et al. 2005).

Activation of polyol pathway resulting from increased aldose reductase activity is one of the mechanisms underlying the development of secondary complications of T2D in diabetic patients. Aldose reductase is the rate‐limiting enzyme in the polyol pathway that reduces excess d‐glucose into d‐sorbitol with concomitant conversion of NADPH into NADP^+^ (Jesus and Sonia 2003). It is present in tissues such as retina, lens, nerve, and glomerulars cells (Ramasamy and Goldberg 2010), where glucose uptake is modulated by glucose transporters independent of insulin. Previous studies provided evidence for the involvement of this enzyme in diabetic complications including neuropathy, retinopathy, nephropathy, and cataract (Ramana 2011), and that its inhibition by plant extracts is a possible therapeutic strategy to ameliorating these complications (Karasu et al. 2012). In this study, *A*.* digitata* leaves extract inhibited aldose reductase, suggesting that it is capable of ameliorating the complications of T2D that are aldose reductase mediated. However, blanching reduced its inhibitory potency.

The inhibitory effect of the extracts on amylase, glucosidase, and aldose reductase could be attributed to the flavonoids and phenolic acids present in them. Previous studies have provided evidence for this; for instance, extracts of *Mangifera indica* and *Mucuna urens* seeds flours rich in these two classes of phenolics inhibited *α*‐amylase, *α*‐glucosidase, and aldose reductase in vitro (Irondi et al. 2014). The decrease in the inhibitory ability of the extract on the carbohydrate‐hydrolyzing enzymes after blanching agrees with that reported by Oboh et al. (2013), who also observed a reduction in the inhibitory ability of A*maranthus cruentus* extract on *α*‐amylase and *α*‐glucosidase activities after blanching. This observation could be a direct effect of the decrease in the phenolics levels, as they are responsible for the inhibition of these enzymes.

## Conclusion

*Adansonia digitata* leaves proved to be a rich source of flavonoids and phenolic acids that are beneficial to health. These phenolics confered antioxidant activity and inhibitory effect against *α*‐amylase, *α*‐glucosidase, and aldose reductase on the leaves extract. Hence, the extract could be effective for the management of T2D. However, blanching reduced the levels of these functional attributes of the leaves, and may not be recommended for their optimal retention.

## Conflict of Interest

We declare that we have no conflict of interest.

## Supporting information


**Figure S1**. I: *Adansonia digitata* (African Baobab) plant; II: Raw *Adansonia digitata* leaves; III: Blanched *Adansonia digitata* leaves.
