# Supplementary material for: Blanching influences the phenolics composition, antioxidant activity, and inhibitory effect of Adansonia digitata leaves extract on α‐amylase, α‐glucosidase, and aldose reductase
Source: Food Sci Nutr. 2016 May 25;5(2):233–42. doi: 10.1002/fsn3.386 (PMC5332274; doi:10.1002/fsn3.386)
Supplement: Supplementary file 1 — Figure S1. I: Adansonia digitata (African Baobab) plant; II: Raw Adansonia digitata leaves; III: Blanched Adansonia digitata leaves. [file FSN3-5-233-s001.doc]

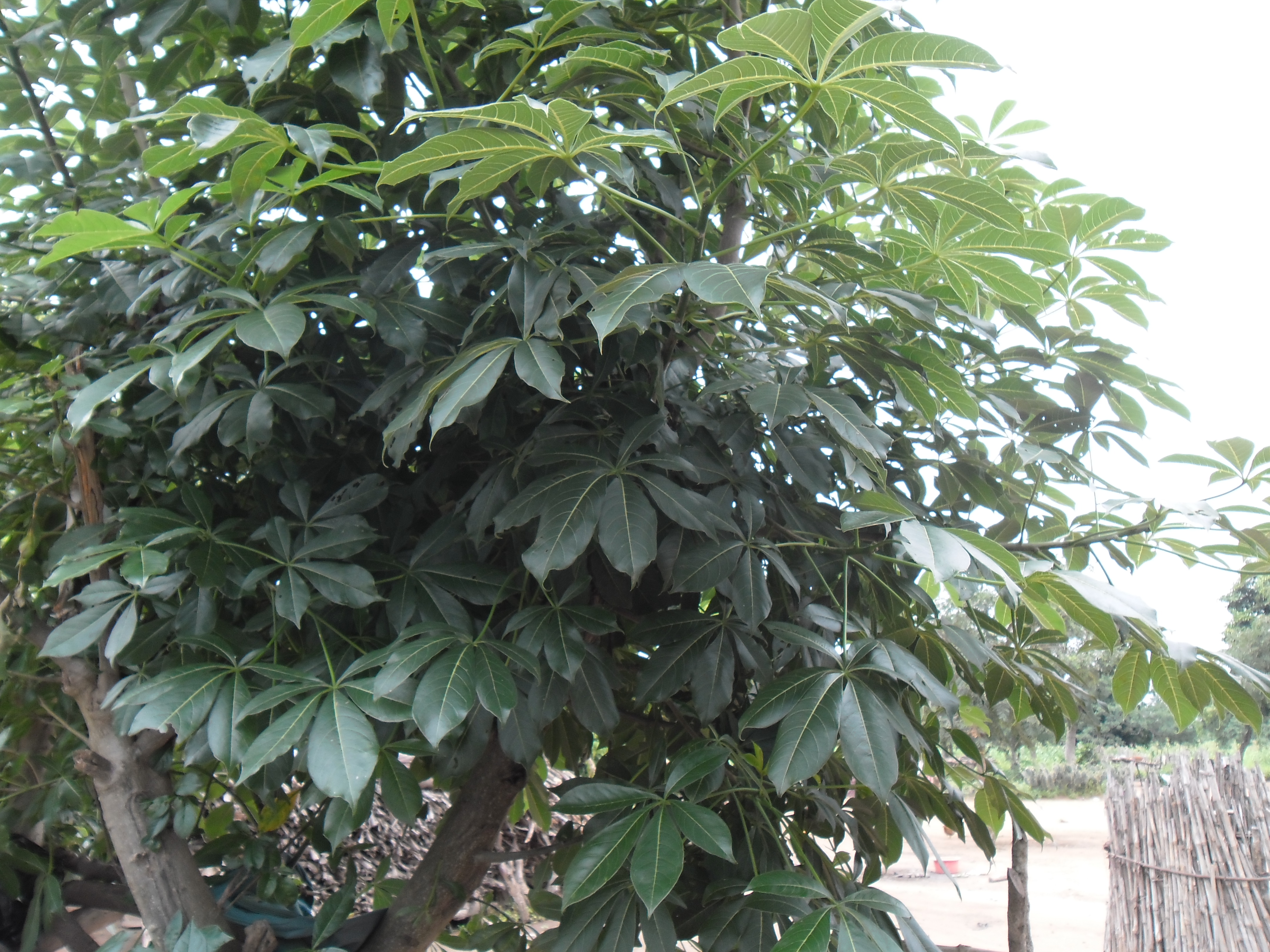


I. *Adansonia digitata* (African Baobab) plant


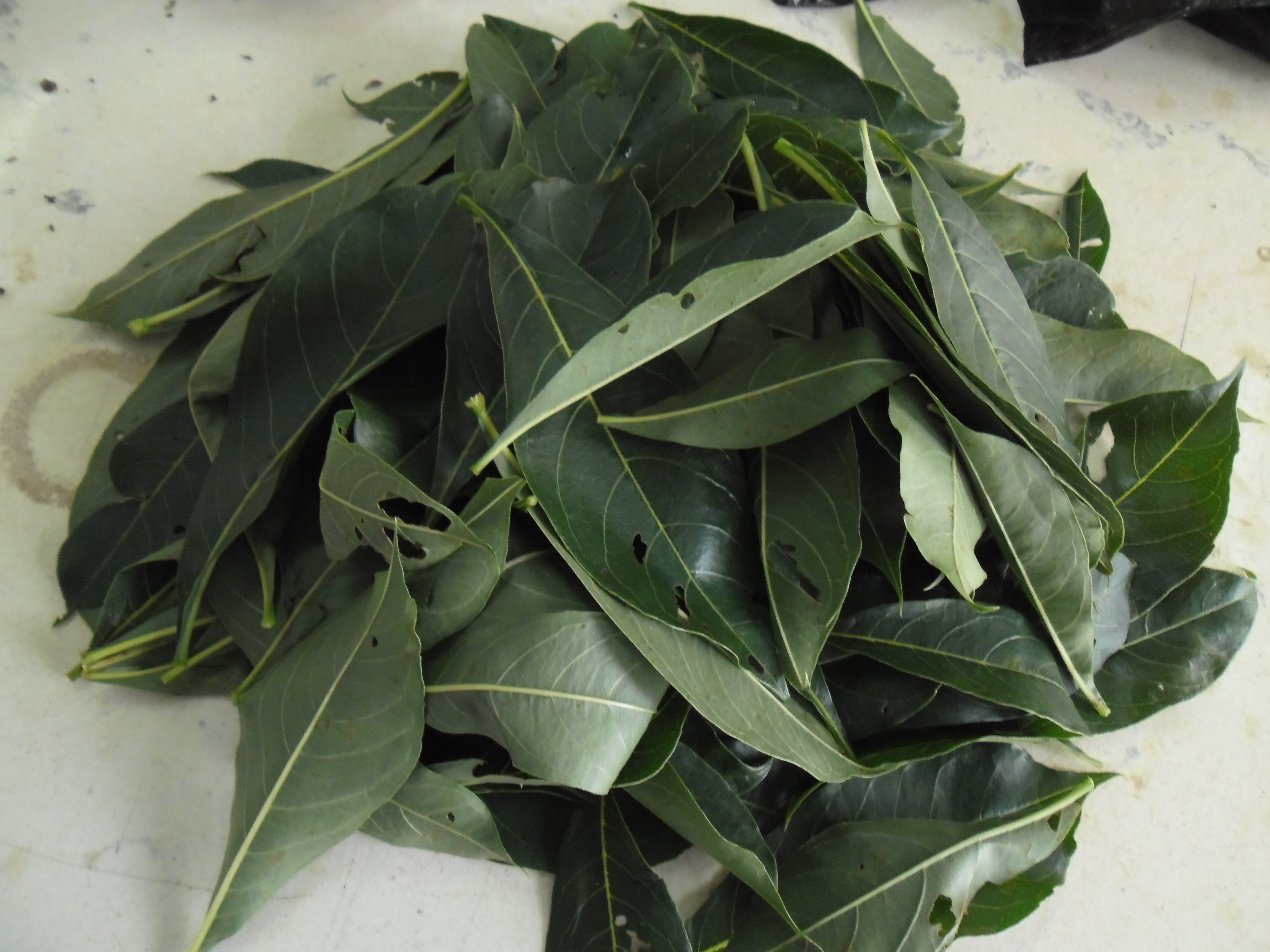


II. Raw *Adansonia digitata* leaves


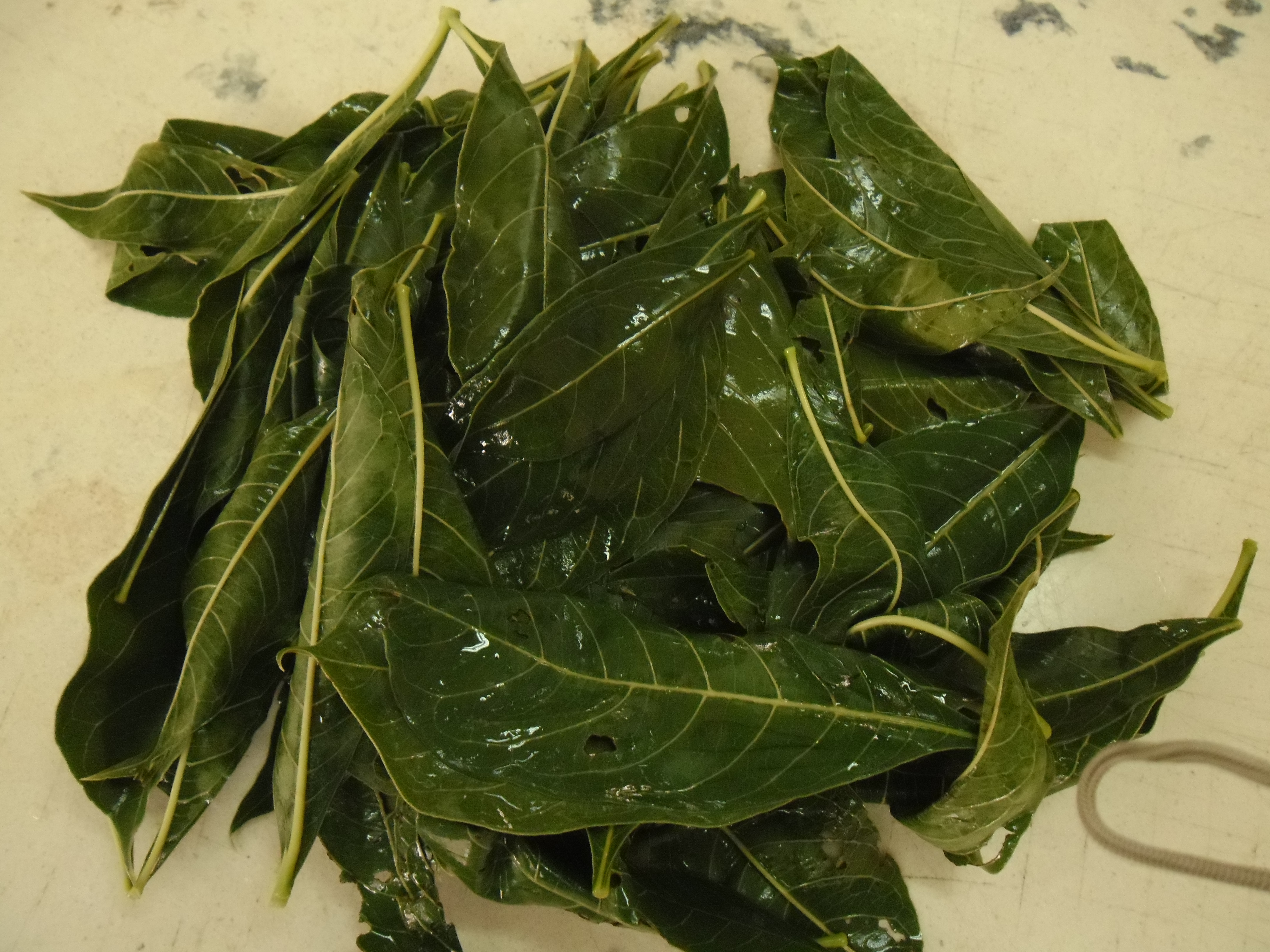


III. Blanched *Adansonia digitata* leaves

Figure: I: *Adansonia digitata* (African Baobab) plant; II: Raw *Adansonia digitata* leaves;

III: Blanched *Adansonia digitata* leaves
